# Oxidative Effects in Early Stages of Embryo Development Due to Alcohol Consumption

**DOI:** 10.3390/ijms25074100

**Published:** 2024-04-07

**Authors:** David González-Flores, Antonia Márquez, Ilda Casimiro

**Affiliations:** 1 Department of Anatomy, Cell Biology and Zoology, Faculty of Medicine and Health Sciences, University of Extremadura, 06006 Badajoz, Spain; 2 Department of Anatomy, Cell Biology and Zoology, Faculty of Sciences, University of Extremadura, 06006 Badajoz, Spain; casimiro@unex.es

**Keywords:** ethanol, FASD, developmental biology, fetus, oxidative stress

## Abstract

Alcohol, a widely consumed drug, exerts significant toxic effects on the human organism. This review focuses on its impact during fetal development, when it leads to a spectrum of disorders collectively termed Fetal Alcohol Spectrum Disorders (FASD). Children afflicted by FASD exhibit distinct clinical manifestations, including facial dysmorphism, delayed growth, and neurological and behavioral disorders. These behavioral issues encompass diminished intellectual capacity, memory impairment, and heightened impulsiveness. While the precise mechanisms underlying alcohol-induced fetal damage remain incompletely understood, research indicates a pivotal role for reactive oxygen species (ROS) that are released during alcohol metabolism, inciting inflammation at the cerebral level. Ethanol metabolism amplifies the generation of oxidant molecules, inducing through alterations in enzymatic and non-enzymatic systems responsible for cellular homeostasis. Alcohol consumption disrupts endogenous enzyme activity and fosters lipid peroxidation in consumers, potentially affecting the developing fetus. Addressing this concern, administration of metformin during the prenatal period, corresponding to the third trimester of human pregnancy, emerges as a potential therapeutic intervention for mitigating FASD. This proposed approach holds promise for ameliorating the adverse effects of alcohol exposure on fetal development and warrants further investigation.

## 1. Introduction

The toxic effects of alcohol, particularly on embryonic development, pose significant public health challenges. It is the main environmental cause of mental retardation and is a preventable cause [1]. Alcohol is a drug; in fact, it is the most consumed drug in industrialized countries. This substance has been linked to human cultures since ancient times, and it was consumed by ancient Egyptians, Greeks, and Romans. However, it is important to consider that alcohol is a drug and one of the main public health problems in industrialized societies [2].

Ethanol is a major component of alcohol. The primary mechanism of ethanol-induced damage is its metabolic process, which produces acetaldehyde and reactive oxygen species (ROS) such as superoxide, hydrogen peroxide, and free hydroxyl radicals. These metabolites contribute to the oxidation of macromolecules, the peroxidation of cellular membranes, the depletion of reduced glutathione (GSH), and ultimately damage to hepatocytes [3,4,5]. Furthermore, interleukin-6 (IL-6) and tumor necrosis factor-alpha (TNF-α) are two inflammatory cytokines that are produced more readily when ethanol and its metabolic products are consumed [6]. Further liver damage is caused by the increased production of certain inflammatory factors, which are partly induced by oxidative stress (OS) and lead to cytokine imbalance and immunological diseases. 

Certain studies have concentrated on cytochrome P450 isoform 2E1 (CYP2E1) [7], a component of the microsomal ethanol oxidation system induced by prolonged alcohol consumption. When ethanol is present, this system has been demonstrated to easily produce ROS, including hydroxyl, superoxide, and hydrogen peroxide [8,9]. It seems that this enzyme, which is involved in the metabolism of ethanol, has reduced activity in the fetus, which leads to the overproduction of ROS [10].

In recent decades, there has been a change in women’s’ consumption patterns; the age of onset has decreased and consumption has increased. In addition, so-called “binges” tend to occur, which are periods of high alcohol intake on weekends or holidays [2].

This widespread alcohol consumption also influences pregnancy. The knowledge that alcohol causes damage during pregnancy is very old. However, it was not until 1968 when [11] conducted a study with children of parents who consumed alcohol during pregnancy and described characteristic facial malformations that a relationship began to be established. In 1973, [12] established alcohol as a teratogenic agent and Fetal Alcohol Syndrome (FAS) was defined.

Although the teratogenic role of alcohol is known, its consumption during pregnancy continues. In studies carried out in different countries (Argentina, Uruguay, Australia, Chile...) during the years 2010 and 2011, it was observed that more than a third of pregnant women consume alcohol during pregnancy, although the majority consume alcohol in small quantities. A small proportion drink alcohol in amounts greater than 40 g/day [2,13]. According to WHO, amounts greater than 10 g/day in women and 20 g/day in men are considered risky consumption. The consumption that is equivalent to 10 g of alcohol is 1 shot, half a glass of wine, or a beer. Around 60% of pregnant women are abstainers; 1% consume more than 40 g of alcohol per day; 2% consume this dose daily; 17% drink moderately (considering a moderate consumption between 20 and 40 g per day); and 20% consume small doses occasionally (less than 20 g) [13,14]. According to the National Survey on Drug Use and Health (NSDUH), 11.0% of pregnant American women used alcohol in the past month [15].

Furthermore, women usually find out that they are pregnant in the second or third month of pregnancy, and this means that the fetus can be unintentionally exposed to alcohol consumption during those first weeks mother. Alcohol consumption occurs more in young people between 20 and 30 years old, the ages at which they are also more fertile and there are more pregnancies.

Alcohol is a substance that crosses the placental barrier by passive diffusion, thanks to its lipid solubility and a low molecular weight that facilitates passage.

The maternal liver takes up most of the alcohol and metabolizes it, but some reaches the fetus, and fetal elimination takes place by hepatic biotransformation and renal excretion. Considering the immaturity of these functions in the fetus and the immaturity of the fetal liver enzymes, ethanol is metabolized slowly, and concentrations remain high for a longer period [16].

Ethanol exposure during critical periods of embryonic development, such as organogenesis, can lead to congenital defects. The most important of those are the defects in the central nervous system (CNS), such as fetal alcohol spectrum disorders (FASD) and neurological development problems in childhood. In addition to the CNS, other organs, such as the heart, are also affected, and there are complications in pregnancy such as preterm deliveries, low birth weight, intrauterine growth restriction, and even spontaneous abortion [16].

The effects produced by alcohol vary and are influenced by the amount of consumption, the type of consumption (it has even been concluded that occasional consumption of large amounts, as is typical of important events, is more harmful to the fetus than the chronic consumption of smaller doses) [13], the stage of embryonic development, and genetic variability [13]. Even so, a safe consumption level has not been established; that is, there is no tolerable amount that does not cause effects, nor is there a period of pregnancy in which consumption is safe. For this reason, it is very important for the prevention of fetal harm not to consume any amount at any time during pregnancy.

The most notable effect of alcohol on the fetus is FAS. This term refers to facial anomalies, delayed growth, and cognitive and behavioral problems that babies present because the mother ingested alcohol during pregnancy [17]. With the definition of this term, alcohol is identified as a teratogen. The identification of this condition established to the starting point for many subsequent investigations and marked the beginning of the prevention of alcohol use in pregnancy.

In addition to all this, it must be considered that alcohol consumption during pregnancy can be associated with the consumption of other drugs or teratogenic substances, as when a mother consumes alcohol during pregnancy, the consumption of other drugs is more frequent [18]. During exposure to teratogenic substances, the baby is also influenced by the home environment or the social environment in which the mother lives during this period because an environment in which teratogenic or toxic substances are consumed by the mother or even the consumption of such substances by other people can affect pregnancy. Parental consumption before pregnancy also has an influence. Several studies have shown that children with parents who consume alcohol are more likely to have microcephaly [19].

## 2. Fetal Alcohol Spectrum Disorder (FASD)

FASD is known as a set of alterations that occur in the embryo as a result of prenatal exposure to alcohol through maternal consumption [20]. These alterations include the following:

- alcohol-related birth defects;
- Alcohol-Related Neurodevelopmental Disorder;
- FAS;
- partial fetal alcohol syndrome;

FAS is the most severe, and the rest are ordered from greater to lesser severity.

FASD is the most common cause of non-heritable intellectual deficits.

For the diagnosis of these conditions, there are no unified criteria. Systems used include the criteria of the Centre for Disease Control and Prevention, the Four-Digit Diagnostic Code, the criteria of the Institute of Medicine of the National Academy of Sciences of the United States, the Washington Criteria of Astley and Warren, and the Canadian criteria, among others [21].

### 2.1. Characteristics of Patients with FASD

Diagnosis begins with an assessment of prenatal alcohol exposure [22]. Exposure is considered high-risk when it meets some of the conditions listed below [23]:

- Six or more drinks per week throughout two or more weeks of pregnancy;
- Three or more drinks per occasion on two or more occasions;
- Legal or social problems related to alcohol during pregnancy;
- Alcohol intoxication during pregnancy registered in blood, lung (air), or urine;
- Positive alcohol biomarker test during pregnancy. Such tests are considered positive when fatty acid ethyl esters, ethyl glucuronic acid, or phosphatidyl ethanol is detected in the hair, nails, placenta, meconium, or blood.
- Increased prenatal risk associated with alcohol use during pregnancy.

Although these are high-risk criteria, any alcohol exposure can pose a risk. There is no safe amount of alcohol for the fetus.

In addition, it is necessary to ensure that there is no exposure to other teratogenic agents because the consumption of other drugs is more likely in women who consume alcohol during pregnancy.

The four categories of FASD are differentiated by the absence or presence of central nervous system dysfunction, dysmorphic facial features, neurobehavioral disabilities, and retarded growth.

### 2.2. FASD Subtypes

The different FASD subgroups are classified according to four categories of abnormalities: (A) growth deficiencies; (B) facial characteristics, (C) abnormalities of the central nervous system, and (D) intrauterine exposure to alcohol [24].

A diagnosis of FAS requires severe abnormalities in all of the first three categories; the diagnosis of this syndrome can be made even without confirmation of prenatal alcohol exposure. Therefore, FAS is defined as a condition in which a child presents with characteristic facial features, prenatal and postnatal growth retardation (percentile < 10) and central nervous system abnormalities with or without certainty of prenatal alcohol exposure.

For the diagnosis of the less severe forms, it is necessary to confirm the consumption of alcohol by the mother during the pregnancy.

In the diagnosis of partial fetal alcohol syndrome according to the Canadian guidelines, there is involvement of all categories except restricted growth; the child will be in a normal percentile (>10).

The diagnosis of alcohol-related birth defects requires the presence of typical facial characteristics and prenatal alcohol exposure without alterations to the central nervous system; a growth deficit may or may not be present.

Lastly, patients with alcohol-related neurodevelopmental disorder do not present with facial dysmorphia, and a growth deficit may or may not be present. The main markers in these children are neurological abnormalities and exposure to alcohol during pregnancy.

## 3. Metabolism of Alcohol

Alcohol is a substance that the human body can metabolize and eliminate; however, its presence in the blood is not trivial, since alcohol is teratogenic, genotoxic, carcinogenic, hepatotoxic, and neurotoxic [25].

The degradation of ethanol, which is present in alcoholic beverages, occurs fundamentally in the liver, where 90% of its elimination is carried out by hepatic oxidation; the remaining 10% can be eliminated by accessory pathways such as the kidneys and the lungs.

**Figure 1:**
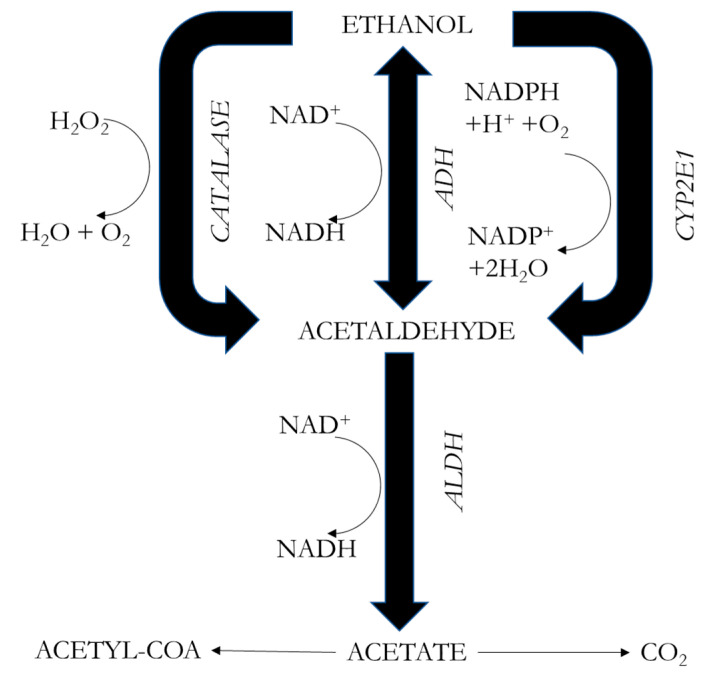
Oxidative metabolism of ethanol. Acetaldehyde is the byproduct of the metabolism of ethanol by several enzymes, including CAT (catalase), CYP2E1 (cytochrome P450, family 2, subfamily E, member 1), ADH (alcohol dehydrogenase), and ADLH (aldehyde dehydrogenase).

Three distinct enzymes are involved in the metabolism of alcohol in the liver (Figure 1), namely alcohol dehydrogenase (ADH); cytochrome P450, family 2, subfamily E, member 1 (CYP2E1); and catalase (CAT) [26,27,28,29]. ADH takes the lead in the liver and stomach, as it metabolizes 90% of ingested ethanol [30]. This enzyme catalyzes the formation of acetaldehyde by transferring the hydrogen from the OH group to the cofactor nicotinamide adenine dinucleotide (NAD) to convert the cofactor to NADH and then, by transhydrogenation, to NADPH. Therefore, it influences the ratio of reduced to oxidized nicotinamide adenine dinucleotide (NADH/NAD^+^) [27]. Women have a lower level of ADH activity than men, so with the same alcohol intake, they have higher blood alcohol levels. This process leads to an imbalance in the mitochondrial respiratory chain, as more electrons enter than can be accommodated by available oxygen molecules, inducing stress in the mitochondria. Consequently, an excess of ROS, including superoxide ions (O_2_^−^) and hydrogen peroxide (H_2_O_2_), is generated [31,32]. Subsequently, acetaldehyde is oxidized to acetate by the enzyme aldehyde dehydrogenase (ALDH), producing an excess of NADH that increases the NADH/NAD ratio. Acetate is ultimately incorporated into the Krebs cycle as acetyl coenzyme A (acetyl–CoA). This acetate can either enter the bloodstream and undergo conversion to carbon dioxide (CO_2_) in the brain, heart, or skeletal muscle, or enter the cytoplasm to form acetyl–CoA [27,33].

The second system involved is the microsomal ethanol oxidation system, in which CYP2E1 plays a major metabolic role in liver microsomes; the transcription of this gene is activated under conditions of high or chronic alcohol consumption, promoting ethanol biotransformation through this pathway. Ethanol is metabolized to acetaldehyde using phosphorylated NAD or reduced NAD (NADPH) and oxygen (O_2_). However, this process also gives rise to ROS such as O_2_^−^ and OH^−^ (hydroxyl radical). This system contributes 3% to 8% of alcohol metabolism but, in the brain, assumes a more prominent role, metabolizing 20% of ethanol [29]. 

The third system is located in the peroxisomes of liver cells, where catalase metabolizes alcohol through peroxidation to acetaldehyde; this is done in the presence of H_2_O_2_, which is then transformed into water. This system metabolizes less than 2% of the alcohol ingested. Notably, in the brain, catalase emerges as the predominant pathway, metabolizing 60% of ethanol [27,29].

This metabolism causes levels of acetaldehyde and hydroxyl radicals to increase in tissues where acetaldehyde can be further oxidized to acetate by acetaldehyde dehydrogenase. This results in an increase in the respiratory chain’s activity, which produces high levels of ROS, which can cause cell damage, lipid peroxidation, and alcohol-mediated inhibition of cell adhesion and can also disrupt growth factors (growth hormone, growth factor insulin-1) [34,35].

An intriguing aspect to consider is the comparison of the causal link between oxidation and FASD as opposed to genetic disorders. Remarkably, individuals with FASD are conceived as initially healthy, with alcohol-induced oxidation catalyzing the pathology by exerting its effects on an otherwise physiologically sound substrate [23,36,37]. Notably, the scientific community has identified a profound impact of alcohol exposure on the hippocampal proteome, where it results in the alteration of over 600 proteins crucial for axonal growth regulation. These proteins include annexin A2, nucleobindin-1, and glypican-4 in the hippocampus and cadherin-13, reticulocalbin-2, and ankyrin-2 in the cerebellum, all of which play pivotal roles in cellular growth and developmental morphogenesis [38].

The challenge posed by alcohol intake during pregnancy lies in the limited or null capacity of the fetus to metabolize and eliminate alcohol. Enzymatic activity gradually increases during gestation, with cytochrome CYP2E1 exhibiting an activity surge from 40% in the second trimester to 80% in the third trimester compared to adults [23]. Consequently, the fetus is likely able to metabolize less than half of the alcohol consumed by the mother [29].

The surge in ROS associated with FASD is attributed to NADPH-dependent NOX enzymes [39], particularly NOX2 and NOX4 [29], which are expressed in microglia, astrocytes, and the cerebral vascular system [7]. Early ethanol exposure during pregnancy is implicated in heightened activity of NOX isoforms, which leads to a substantial increase in ROS, cellular damage, and eventual apoptosis in FASD patients [29]. This pathway, coupled with the reduced detoxification activity of fetal CYP2E1, appears to explain the drastic rise in ROS and the resultant phenotype of FASD patients [40].

Although a comprehensive description of the alcohol-induced mechanisms of neuropathology in vulnerable regions remains elusive, the teratogenic effects are believed to stem from ethanol-induced dysregulation of various intracellular pathways, which culminates in toxicity and cell death [41]. Ethanol exposure induces an imbalance in the intracellular redox state, generating an overall increase in OS that is particularly impactful in the brain due to its high content of fatty acids—a perfect substrate for ROS [42]. The enzymatic activity of CYP2E1, coinciding with brain organogenesis, oxidizes ethanol, generating hydroxyethyl or superoxide radicals that target polyunsaturated fatty-acid side chains in brain-tissue membranes [38,40]. Consequently, fetal brain tissue sustains damage during organogenesis, resulting in neurological dysfunctions post-birth [7,40,42,43].

### 3.1. Changes in Metabolism and Blood Alcohol Concentration during Pregnancy

Alcohol crosses the placenta by passive diffusion, thus exerting toxicity on the fetus. The damage that this consumption can produce depends on the dose, the duration, and the stage of embryonic development in which the exposure occurs. The first trimester is a stage of high vulnerability to these teratogenic substances because organogenesis occurs between days 18 and 60 of gestation [44].

In addition, during pregnancy, there are physiological changes that alter the kinetics of drugs, increasing toxicity and the risk of complications, as follows [45]:

- The absorption of alcohol varies during pregnancy because in this state there is a delay in stomach emptying and a decrease in intestinal motility. Therefore, it can be expected that in pregnant women, the concentrations of alcohol in the blood will be maintained for a longer time, although the serum alcohol peaks will be lower.
- Alcohol, once in the blood, passes to the placenta by simple diffusion. The diffusion balance is based on the water content. During pregnancy, the volume of water increases up to 6 L in all compartments, including the amniotic fluid, the placenta, the uterus, and the fetus. During the different stages of pregnancy, there are some changes in the volumes of water. Therefore, fetal exposure to alcohol varies according to the changes in the amounts of water in the maternal and fetal organisms. During early pregnancy, the fetal water level is very high, and therefore the fetus is highly exposed to alcohol.
- Alcohol is eliminated through oxidation by ADH in the liver. However, the fetus’s liver enzymes do not mature until about the second half of pregnancy, so it cannot metabolize alcohol. The alcohol concentration in the maternal body is more correlated with fetal harm than is the total amount of alcohol administered.

### 3.2. Markers of OS during Pregnancy

Some studies have elucidated the main molecular markers in ethanol users [46]. Table 1 presents the OS marker results for the ethanol and control groups. The Alcohol Use Disorder Identification Test (AUDIT) median was computed to assess the impact of alcohol consumption on OS indicators. As a result, two groups of alcohol users were created: AUDIT lower than 24.5 and AUDIT greater than 24.5 (Table 2). Malondialdehyde (MDA), a stable and membrane-permeable molecule, is the end product of the breakdown of polyunsaturated fatty acids. It can form adducts with proteins and DNA and is thus associated with various pathological conditions, including Alzheimer’s disease, Parkinson’s disease, cardiovascular and liver diseases, diabetes, and cancer [47]. The group exhibiting chronic alcohol consumption demonstrated elevated concentrations of MDA and SOD compared to the control group. Consistent with the existing literature [48,49,50], MDA levels were notably higher in alcohol drinkers, suggesting OS-induced damage in comparison to the control group. However, conflicting findings emerged concerning the enzymes responsible for ROS elimination. Another finding of that study was a negative association between the ferric reducing/antioxidant power (FRAP) and alcohol intake; that is, Table 3 shows that FRAP decreases with increasing alcohol use (higher AUDIT).

Studies utilizing the TBARS method to assess MDA activity consistently reported elevated levels in alcohol users [49,51,52,53,54,55]. While some studies noted increased MDA activity without statistical significance [56,57,58], evaluations of red blood cells consistently revealed higher levels in alcohol users [50]. 

Research indicates a positive impact on MDA activity after a detoxification period of two weeks, leading to reductions in some individuals [48,54] and no significant difference from the control group in certain cases [51,52]. However, contradictory findings from [58] and [59] indicate no alteration in this biomarker before and after dependence treatment.

The SOD enzyme, which is responsible for converting O_2_^−^ into H_2_O_2_ [60], exhibited higher plasma concentrations in the alcohol group, suggesting a compensatory mechanism to eliminate potential excess O_2_^−^. This finding aligns with similar results from previous studies [48], although some reported decreased concentrations in alcohol users [51,52,55] and others found no significant difference between groups [58,61]. Studies assessing SOD erythrocyte activity also presented conflicting outcomes, including higher activity in the alcohol group [49,62], lower values in alcoholics [53,54], and no significant differences [56,63].

Detoxification, in certain studies, did not induce significant alterations in SOD concentrations [51,52,58,59], while others observed decreased concentrations compared to values at hospital admission [48,61]. The varied outcomes underscore the complexity of the interplay between alcohol consumption, OS markers, and the impact of detoxification interventions.

## 4. Toxicity of Alcohol

The mechanism of ethanol toxicity is not yet established, but there are several hypotheses about it, as follows:

- In 1981, Henderson et al. [24] began to talk about the mutagenicity of ethanol.
- Ten years later, Michaelis discussed the role of interactions between the hypoxic conditions generated by ethanol in the fetus and the mechanisms activated by neurotransmitters in the production of cell damage in developing neurons, as well as abnormalities in calcium-manipulation mechanisms and their effects on migration and neuronal differentiation [25]. It is important to note that modifications in calcium signaling have been related to cell death and apoptosis [35,64,65].
- The latest hypotheses deal with neural death during forebrain maturation due to the blockade of the NMDA-glutamate receptor and the activation of GABA-A receptors [26]. Excitatory amino acids (glutamate) influence the processes of neuronal differentiation and synaptogenesis because they can modulate the organization of neuronal circuits and can opportunely regulate biochemical events related to the phenomenon of neuronal plasticity. Therefore, it is conceivable that if ethanol exposure reduces glutamatergic transmission at critical stages of development, this may play a key role in determining the neurotoxic effects of alcohol abuse.

Alcohol disrupts development through various genetic, epigenetic, molecular, cellular, and physiological pathways.

Both ethanol and its metabolites produce the adverse effects. Ethanol and its metabolite, acetaldehyde, cross the placenta and accumulate in fetal blood. In addition, the embryo does not yet have the enzymes for biotransformation, so they remain in fetal blood for longer.

The organ most affected in the fetus by alcohol consumption is the brain. There is evidence that exposure to ethanol during brain development causes both structural and neurobehavioral alterations. However, the mechanisms by these alterations are produced are complex and not well known.

Several studies have investigated the damage that alcohol produces in an already-developed brain. Macroscopically, MRI studies in adult individuals exposed to alcohol have shown volume loss and loss of myelination throughout the brain [66].

These radiological investigations have also been carried out to investigate prenatal alcohol exposure. The first studies carried out in this regard found a thinning of the corpus callosum with an enlargement of the ventricles, as is characteristic of exposure. However, the most recent research highlights considerable variability in the impact of alcohol on the developing brain, establishing an effect on the brain macrostructure; quantitative analysis shows a lower volume of both grey and white matter [67]. This finding suggests that CNS development is non-selectively inhibited. There is neuronal loss in both the cortex and the cerebellum, hippocampus, and white matter of the brain [68,69]. Lesions in the white matter, demyelination, decrease in oligodendrocytes, and aberrations in these have been observed [70].

Collectively, these results lead to the fact that these findings can be used as markers to help in the diagnosis of these disorders by non-invasive methods such as magnetic resonance imaging [71].

### 4.1. Main Mechanisms of Alcohol Toxicity

There is nothing established about the mechanisms by which alcohol produces alterations in the development of the fetus, especially in fetal brain development. Diverse and complex mechanisms are known. Among them, the two that have been most studied and that have been seen to be most related to alcohol toxicity are neuroinflammation and OS.

#### 4.1.1. Neuroinflammation

As previously stated, this is one of the most developed and important mechanisms that explain alcohol toxicity in the brain.

Alcohol alters the functions of both neurons and glial cells. The latter are responsible for protecting the cells of the CNS and maintaining its homeostasis. Therefore, impaired glial cell function will also damage neurons.

Glial cells as a whole are responsible for removing toxic molecules and cellular debris, regulating energy balance, promoting neurotransmission, and modulating synaptic activity. However, each type also has a more specialized function. Microglia are mainly in charge of mediating the immune response and have a function like those of macrophages and peripheral lymphocytes. Microglia activation occurs to eliminate pathogens and toxic substances through phagocytosis, the generation of cytokines, and antigen presentation. However, chronic activation of these cells can also cause damage to cells of the CNS. Astrocytes are responsible for synapse control and control of energy balance, ions, and neurotransmitters; maintaining the blood-brain barrier; and repairing the nervous system. Oligodendrocytes myelinate neurons and facilitate the transmission of electrical impulses [72].

**Figure 2:**
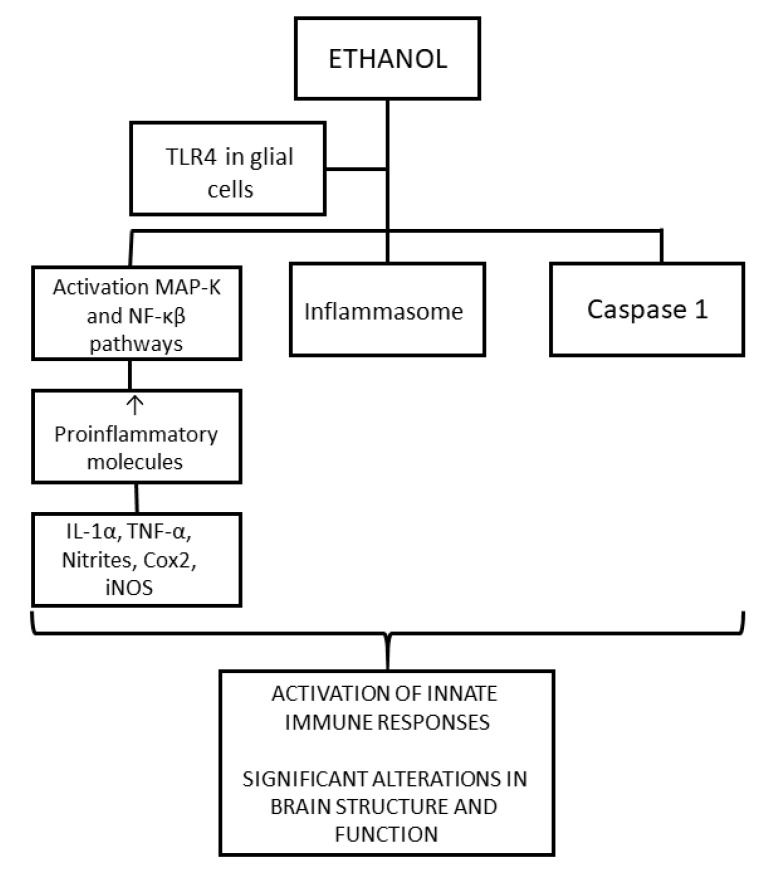
Ethanol produces activation of the immune system. ↑: Increase.

As seen in Figure 2, ethanol activates the innate immune system through the toll-like receptor 4 (TLR4) signaling response in glial cells, producing an increase in the expression of the proinflammatory molecules IL-1α, TNF-α, nitrites, cox2, and iNOS by activating the MAPK and NF-κB signaling pathways. This inflammation may cause damage to neuronal cells. The activation of the innate immune system eventually produces an alteration in myelin proteins, as well as activation of apoptotic proteins, which consequently result in significant alterations in both the structure and the function of the developing brain. It has been verified that these mechanisms depend on TLR4 because this activation of glial cells did not occur in TLR4-knockout mice [25,66,71]. Furthermore, it has been widely found that TNF-α is an important activator of apoptosis in immune cells [65,73].

Ethanol also produces an increase in the production of ROS and decreases the expression of the antioxidant glutathione. Other pathways that also lead to an increase in proinflammatory molecules are the increase in levels of the NLRP3 inflammasome and the activation of caspases, especially caspase 1, as caspase 1 is a proinflammatory molecule.

All these pathways produce an increase in pro-inflammatory molecules in the brain, an immune response, and activation of glial cells, especially microglial cells, which can lead to apoptosis of neurons and alteration of glial cell functions. Neuronal plasticity and the survival of neurons are altered, and there are defects in myelination (demyelination) and alterations in the conduction of nerve impulses, explaining the cognitive and behavioral impairments and memory alterations present in patients with FASD.

#### 4.1.2. Oxidative Stress

OS is the main pathway of ethanol damage. This pathway affects both the brain and other organs of the developing fetus.

ROS can be generated by mitochondrial respiratory chain enzymes, xanthine oxidase, cytochrome P450 (one type is 2E1, CYP2E1), and NADPH oxidase (NOX). Alcohol generates ROS via CYP2E1 and NOX.

As previously seen, ethanol is metabolized to acetaldehyde via various pathways, specifically ADH and CYP2E1 (to a much lesser extent also via CAT); however, the CYP2E1 enzyme produces ROS as by-products. OS is generated by ethanol and ROS due to excessive production of NADH and the need to balance the NAD/NADH ratio through lactate dehydrogenase.

In an uncontrolled manner, ROS oxidizes proteins, lipids, and other metabolites, causing DNA damage [35,64,65,73]. An increase in DNA damage activates apoptosis pathways that lead to neurodegeneration [74].

Serotonergic neurons are especially susceptible to ethanol-induced apoptosis. These apoptosis events have been correlated with abnormalities of cortical structures and decreased brain volume, which lead to alterations in behavior and cognition [75].

Both the adult and fetal brain are the main targets of ROS and OS. The reason that brain tissue is especially sensitive is multifactorial. High levels of ROS are implicated, along with the fact that the brain is the tissue with the highest oxygen metabolic rate, and is rich in autoxidizable neurotransmitters and unsaturated fatty acids that are ROS substrates. It is also pertinent that the reactions with ROS generate superoxides, quinones, and semiquinones that again are highly reactive radicals. Levels of antioxidant enzyme (SOD, CAT, GPx) are lower in the brain than in other tissues. All these factors make the brain tissue especially susceptible to ROS reactions produced by alcohol. In addition, it must be considered that fetal cells are more exposed to ethanol because they have fewer ethanol-degrading enzymes and because ethanol remains longer in fetal blood [18,76].

Animal studies have shown that the application of antioxidants rescues some phenotypes induced by ethanol toxicity [67]. Furthermore, the novel study of nanomaterials has shown them to have potential as an antioxidant therapy [65]. However, in humans, this conclusion is questionable.

Cell death in selected cell populations has been observed to be a defining pathologic feature of FASD [77]. There is increasing evidence that OS plays an important role in teratogenesis and ethanol-induced apoptosis.

There is a relationship between these mechanisms. In neurodegenerative diseases, the presence of neuroinflammation and OS is characteristic, and a relationship between the two has been detected, and although these studies have not been carried out to examine FASD, they could be extrapolated to this condition [78].

It has been shown that microglia activation and ROS generation are mutually related in the neuroinflammation process. ROS act as mediators in the activation of microglia and the consequent activation of the immune system, activating MAPK, NF-κB, and the NLRP3 inflammasome [79]. ROS are not the main trigger of neuroinflammation but would be secondary messengers of microglia, thus contributing to the immune and inflammatory dysregulation that occurs. On the other hand, microglia generate ROS, activating NOX, to eliminate dead neurons and cells and aggregated proteins.

#### 4.1.3. Other Mechanisms of Teratogenesis and Alcohol Toxicity

As previously stated, there are other mechanisms that have been found to be involved in alcohol-induced damage, although without a clear relationship, as follows:

- Neurotrophins [19,80];
- Ischemia or hypoxia;
- Thiamine deficiency [66,81];
- Dysregulation of mitochondrial bioenergetics [76];
- Epigenetic alterations of alcohol in the cells of the nervous system [82,83,84,85,86].

In conclusion to this section, the evidence from experimental studies carried out in animals and observational studies in humans shows that prenatal alcohol exposure may interrupt the normal processes of fetal development through a multitude of mechanisms that include the interruption of cellular metabolism (i.e., OS, protein suppression, and DNA synthesis), impaired cell growth, altered gene expression, interference with growth factor signaling, increased cell damage and apoptosis, and altered placental function and hemodynamics, which result in fetal hypoxia [86].

## 5. Influence of Genetics on the Variability of the Disease

Genetics plays an important role in FASD. The effects of prenatal exposure to alcohol are mediated by susceptibility factors, which explain the different presentations of FASD and the different responses to the same amount of alcohol consumed. Different sensitivities to FASD correlate with different isoforms of alcohol-degrading enzymes and different ADH alleles. ADH1B variants process alcohol more quickly, and there are fewer cases of FASD associated with this variant; on the other hand, an ADH1C allele is associated with facial clefts in alcohol-exposed children. SMC2 and MLLT3 are strongly associated with alcohol-induced facial clefting [87]. 

ADH variations can result in increased acetaldehyde levels, increasing levels of ROS, altering the balance between antioxidants and pro-oxidants, and damaging DNA. Loss of both ADH and the DNA repair enzyme from the Fanconi anemia gene family results in alcohol-induced encephalopathy and ocular defects. In addition, alcohol may also be broken down by retinaldehyde dehydrogenase (RALDH). RALDH converts the retinal into retinoic acid, which is essential for the development of many systems and organs; alcohol is a competitive inhibitor of retinoic acid (RA) synthesis. Retinoic acid supplementation may prevent alcohol-induced defects [87].

In communities with varying ancestries, the impact of common ADH variants has been investigated. Various variants have demonstrated some protective effects in various populations. Other variants relating to growth and nutrition metabolism have been associated by candidate gene studies in smaller cohorts with the outcome of FASD; in an investigation based on genome-wide association studies, single-nucleotide polymorphisms linked to alcohol-use disorder showed some relationships with drinking habits during pregnancy [88]. 

A total of 428 illnesses were identified in a recent study as co-occurring with FASD, and the most frequently occurring conditions were linked to chromosomal abnormalities, congenital malformations, and deformities [89]. These abnormalities include ring chromosome 6 and trisomy 21, both of which have been linked to FASD in earlier research [90,91,92]. Attention deficit is one of the most prevalent phenotypes linked to FASD, and children with FASD have up to a 10-fold higher prevalence of attention-deficit hyperactivity disorder [93].

Therefore, genetic factors are central to the impact of alcohol.

## 6. Pharmacological Treatments under Study

No pharmacological treatments can be used in humans, but there are several lines of research underway to discover future lines of treatment based on inhibiting the mechanisms of toxicity produced by ethanol. Among them are antioxidants and anti-inflammatories.

The NADPH-dependent enzymesNOX2 and NOX4 [94,95] are members of a family of proteins expressed in brain arteries, astrocytes, microglia, and neurons that generate ROS. In FASD, they are overexpressed [10]. ROS inhibitors, such as diphenyleneiodonium NOX, which decreases ethanol-induced apoptosis, prevent significant increases in NOX enzyme activity, ROS generation, and oxidative DNA damage [77].

Metformin, a widely used insulin-sensitizing drug, enhances short-term memory [96] and aids in the formation of spatial memories [97]. Additionally, it is a promising treatment, as it provides neuroprotection against ethanol toxicity [98]. This neuroprotection is produced by its antioxidant and anti-inflammatory activities. A decrease in OS is produced by increasing the activity of the antioxidant system of glutathione and the antioxidant enzymes CAT and GSH. It also performs anti-inflammatory functions by decreasing the level of TNF-α.

Metformin has demonstrated notable effects on memory performance in mouse models of Alzheimer’s disease, as evidenced by studies such as that by Farr et al. [99]. In addition to its cognitive benefits, metformin significantly lowers the risk of Parkinson’s disease in diabetic patients and protects against injuries related to 1-methyl-4-phenyl-1,2,3,6-tetrahydropyridine, as indicated by research conducted by Patil et al. [100]. Metformin’s multifaceted impact extends to the reduction of ROS and ROS-induced DNA damage through the suppression of mitochondrial respiration [101]. Furthermore, it plays a crucial role in safeguarding the antioxidant defense system and elevating glutathione levels, as highlighted in studies such as that by Fang et al. [102]. 

The persistent activation of microglia and astrocytes leads to the production of proinflammatory and neurotoxic factors, including IL-1β and TNF-α, which contribute to neurodegeneration by triggering apoptotic cell death in vulnerable neurons [103,104]. Proinflammatory cytokines can elevate ROS concentration by affecting uncoupled eNOS, NADPH oxidase, and mitochondrial respiratory chain complexes [105]. The interaction between TNF-α and TNF receptor-1 initiates signal transduction pathways, forming a multiprotein signaling complex in the cell membrane. Subsequently, in neurons, proinflammatory molecules activate downstream apoptotic signaling pathways, leading to the activation of glial cells, neuronal death, and the promotion of neuroinflammation [106].

A study showed that treatment with metformin significantly attenuated the ethanol-induced decrease in antioxidant enzyme concentrations (Table 4) [107]. Considering these findings, metformin, with its anti-inflammatory properties, emerges as a potential therapeutic agent capable of mitigating changes in TNF-α levels following ethanol-related neurotoxicity in the hippocampus.

Abnormal thyroid function frequently accompanies dysfunctional glucose metabolism [108]. In both ethanol-consuming dams and their adult offspring, there is evident hyperglycemia with no discernible changes in insulin levels, implying insulin resistance [109,110]. This condition necessitates an increased release of insulin from the pancreas to maintain normal plasma glucose levels. Considering the association between insulin-pathway genes such as insulin-like growth factor 2 (Igf2) and hippocampus-based learning and memory processes [111,112,113,114], peripheral and central insulin resistance may contribute to fetal alcohol exposure (FAE)-induced learning and memory deficits [115]. The reduction in Igf2 expression during development, a consequence of FAE [116,117], is known to be detrimental to cognition [118]. Normalizing Igf2 expression could potentially reverse FAE-induced cognitive deficits [119]. Metformin not only provides neuroprotection against ethanol-induced neurodegeneration but also influences Igf2 expression. Consequently, metformin emerges as a logical candidate for exploration as a potential treatment to counter FAE-induced memory deficits [120].

Dio3 and Igf2, both imprinted genes, typically exhibit paternal allele expression in the placenta [121,122]. However, in the adult hippocampus, they show preferential maternal expression [123,124]. The shift from paternal to maternal expression is not fully understood, but it is known that imprinting, in general, is regulated by differential DNA methylation of the maternal and paternal alleles [125]. As FAE alters DNA methylation [117,126,127,128], the changes in allelic expression of Dio3 [129] and Igf2 [116] likely result from modifications in DNA methylation at these imprinted loci [130]. DNA methyltransferase 1 (Dnmt1) [125] and enhancer-blocker CCCTC-binding factor (Ctcf)-binding sites play analogous roles in the maintenance and regulation of DNA methylation in the Dio3 and Igf2 imprinted regions [131]. If FAE affects DNA methylation and the imprinting of these genes similarly, neonatal T4 or metformin treatment could potentially reverse the effects of FAE on these imprinted genes and their common regulators [120].

The work [120] aimed to assess the effects of neonatal administration of T4 or metformin on adults with FAE as part of a search for potential treatments for FAE-induced cognitive deficits. The treatment period was post-FAE, corresponding to a neurodevelopmental stage equivalent to the third trimester of human pregnancy. Successful treatment during this period enhances the translational value of the study, as some women may reduce alcohol consumption toward the end of pregnancy [132]. Treating women is more feasible than treating newborns, and neurodevelopment can still be affected. The secondary objective was to determine whether neonatal treatment with T4 or metformin could normalize hippocampal expression of Dio3 and Igf2. Neonatal rats received T4 and metformin during postnatal days 1–10 after the maternal consumption of alcohol ceased. 5-Aza, a Dnmt inhibitor that preferentially inhibits Dnmt1, was also given to control neonates as a proof of concept for the role of Dnmt1 in FAE-induced memory loss. Table 5 shows a summary of the results. Additionally, both male and female offspring were investigated, reflecting reported sex differences in the effects of FAE in both human [133] and animal studies [134,135]. The hypothesis posited that altered maternal thyroid and glucose functions due to alcohol consumption create an adverse intrauterine environment for offspring development, and administration of T4 and metformin within a specific neonatal developmental window may reverse these adverse effects.

Recent meta-analyses and trials indicate that metformin shows promise in preclinical models for reducing oxidative stress induced by prenatal alcohol exposure, yet clinical trials in humans remain limited [136,137].

There are other pharmacological approaches currently being evaluated. For example, in a recent study using a mouse model, a diet rich in folate and choline shielded methylation of the mother’s DNA from some of the negative consequences of early, moderate prenatal alcohol exposure. These findings point to a role for preventive maternal dietary interventions and show that even in the absence of overt phenotypic alterations, early moderate exposure is enough to impact fetal genome regulation [138].

Future research should explore the potential of targeted antioxidant therapies beyond metformin, especially those that can cross the placental barrier and directly mitigate oxidative stress in the developing fetus.

## 7. Discussion

Throughout history, alcohol consumption in pregnancy has been linked to harm. However, it was not until relatively recently, in 1973, that the disorders and effects it produced began to be defined and investigated [12].

The quintessential manifestation of alcohol use disorder in pregnant women, fetal alcohol spectrum disorder, presents well-defined characteristics: facial dysmorphia, growth retardation, and central nervous system involvement, the latter expressing itself as neurobehavioral alterations. However, alcohol also produces other effects on different organs that are not so clear.

Alcohol produces its toxicity by various mechanisms, many of which are still unclear. The main mechanisms are the following:

- Oxidative stress: Ethanol metabolism produces ROS that cause lipid peroxidation, protein oxidation, and DNA damage. It is well known that these events produce apoptosis and neurodegeneration [35,64]. Within the context of pediatric disorders, such as FASD, OS may have a minor first-order effect [10]. Preclinical research has demonstrated that alcohol consumption during pregnancy impairs the capacity of potassium channels to dilate cerebral arterioles; this disruption appears to be mediated also by elevated levels of OS [139]. Additionally, in a rat model, neonatal ethanol exposure causes deficiencies in context-dependent fear learning and depressive-like behavior, which are linked to higher levels of OS in the hippocampus and prefrontal cortex [140].
- Neuroinflammation: Ethanol activates the innate immune system in the fetal brain by activating TLR4, producing cytokines and proinflammatory molecules. This activation produces gliosis, neuronal inflammation, alteration of myelin, and neuronal damage.

In neurodegenerative diseases, these mechanisms are related; relevant studies have not been carried out to examine FASD, but the results of other studies can be extrapolated. TLR4s activate NOX to produce ROS and thus facilitate the removal of damaged cells and other products. ROS activate the MAPK and NF-κB pathways to contribute to inflammation and activation of the immune system.

The study of these mechanisms is very important to developing treatments for mothers who consume alcohol and children with FASD because currently, the only measure available is prevention. Antioxidants are under investigation as treatments, as are ROS inhibitors such as diphenyleneiodonium, which decrease ethanol-induced apoptosis; anti-inflammatories; nanoparticles; and metformin, which is a promising treatment with anti-inflammatory and antioxidant properties that should decrease apoptosis and inhibit the mechanisms of toxicity of alcohol in the brain. These studies have shown efficacy in animals, but there is still a long way to go before it can be used in humans [67,141].

Despite improvements in pharmacology, the first and most important step in reducing the effects produced by alcohol consumption in the early stages of embryonic development is the prevention of alcohol consumption during pregnancy. It all starts with good health education for the youngest generations regarding the damage that alcohol can cause both in the adult and the fetus. Recommendations for pregnant women, as well as social and psychological support for the most vulnerable groups, are required [142]. 

Although FASD is preventable, it remains highly prevalent worldwide. Therefore, an effort must be made to prevent it. FASDs result in educational, medical, and social repercussions in all stages of life for individuals who suffer from them and for the people around them. However, there is a shortage of interventions in adults and adolescents, and the lack of adequate interventions for these age groups promotes the development of secondary disabilities. Therefore, there is a great need to share and implement appropriate contextual/cultural interventions for the prevention and management of FASDs [143].

It could be concluded that there is still much to investigate regarding this topic. Future research is necessary to clarify the effects produced by alcohol consumption, the mechanisms of toxicity, and the treatments and action plans necessary after diagnosis. the results of these investigations can prevent a large number of cases. These investigations are also necessary because an increase in the incidence is expected. Even though more research is required to fully comprehend the connection between OS and pediatric illnesses, understanding this important topic now can help shape treatment approaches in the future.

It is necessary to research the effects that alcohol produces because the fact that alcohol is harmful to the fetus is generally known but the effects of consumption are not and because there are doubts about the amount of alcohol that is harmful. It is necessary to clarify these aspects and insist on the consumption of zero alcohol to reduce the prevalence of these conditions. It is also important that professionals consider these disorders to promote prevention and early diagnosis. 

## Figures and Tables

**Table 1:** Markers of oxidative stress in users and nonusers of alcohol. CAT: catalase; FRAP: ferric reducing/antioxidant power; GPx: glutathione peroxidase; MDA: malondialdehyde; SOD: superoxide dismutase. Data are reported as median (U/I). * *p* < 0.05. Mann Whitney U test [46].

Enzyme	Control	Alcohol	*p*
CAT	1.67	0.95	-
FRAP	1143.14	1344	-
GPx	23.07	6.9	-
MDA	1.25	1.47	*
SOD	93.9	433	*

**Table 2:** Markers of oxidative stress in low (AUDIT < 24.5) and high ≥ 24.5) alcohol users. CAT: catalase; FRAP: ferric reducing/antioxidant power; GPx: glutathione peroxidase; MDA: malondialdehyde; SOD: superoxide dismutase. Data are reported as median (K/s). * *p* < 0.05. Mann–Whitney U test [46].

Enzyme	Low	High	*p*
CAT	0.56	1.12	*
FRAP	1515	1292	-
GPx	12.34	4.07	-
MDA	1.81	1.37	-
SOD	533	417	-

**Table 3:** Correlation between markers of oxidative stress and AUDIT. OS: oxidative stress; SOD: superoxide dismutase; CAT: catalase; GPx: glutathione peroxidase; FRAP: ferric reducing/antioxidant power; MDA: malondialdehyde. * *p* < 0.05, Spearman’s correlation [46].

Enzyme	Spearman’s Correlation	*p*
CAT	0.167	-
FRAP	−0.299	*
GPx	−0.188	-
MDA	0.065	-
SOD	0.033	-

**Table 4:** Effect of metformin administration after the induction of ethanol-related neurotoxicity in the hippocampus. GPx: glutathione peroxidase activity; MDA: MDA concentration; Met20: treatment metformin (20 mg/kg); Met40: treatment metformin (40 mg/kg); SOD: superoxide dismutase activity; TNFα: TNFα concentration. ↑ represents an increase and ↓ represents a decrease [107]. 1 arrow = low decrease/increase, 2 arrows = medium decrease/increase, 3 arrows = high increase/decrease.

Treatment	SOD	GPx	MDA	TNFα
Milk + Saline	-	-	-	-
Ethanol	↓↓	↓↓↓	↑↑↑	↑↑↑
Ethanol + Met20	↓↓	↓↓	↑↑↑	↑↑↑
Ethanol + Met40	↓	↓	↑	↑

**Table 5:** Effects of metformin and thyroxin on hippocampal allelic expression. ↑ represents an increase and ↓ represents a decrease [120].

Treatment	Ctcf	Dio3	Dnmt1	Igf2
FAE + metformin	↓	↓	↑	↑
FAE + T4	-	↓	↑	↑
5-Aza + metformin	↓	↓	↑	↑

## Data Availability

Data is contained within the article.
